# Hyperuricemia and intravenous fat emulsion are risk factors for gout flares during active gastrointestinal bleeding: a case control study

**DOI:** 10.1186/s42358-024-00376-w

**Published:** 2024-05-03

**Authors:** Yujie Jiang, Xuelian Hong, Bingtian Xia, Hongwei Du

**Affiliations:** 1 Department of Rheumatology, Jinhua Hospital of Zhejiang University: Jinhua Municipal Central Hospital, Jinhua, 321000 China; 2 Department of Hematology, Jinhua Hospital of Zhejiang University: Jinhua Municipal Central Hospital, Jinhua, 321000 China

**Keywords:** Gout flare, Gastrointestinal bleeding, Hyperuricemia, Intravenous fat emulsion

## Abstract

**Objective:**

It is well-established that patients with a history of gout are more susceptible to experiencing gastrointestinal bleeding. Gout flare during active gastrointestinal bleeding poses a significant challenge due to the gastrointestinal side effects of anti-inflammatory therapy. This study sought to investigate the risk factors associated with gout flares during episodes of gastrointestinal bleeding.

**Methods:**

We conducted a retrospective observational study involving 94 patients who experienced active gastrointestinal bleeding and had a history of gout. This study was conducted at Jinhua Municipal Central Hospital from January 2019 to October 2022. We collected and recorded demographic information and clinical characteristics.

**Results:**

Among the gout flare patients, hyperuricemia and intravenous fat emulsion therapy were more prevalent compared to those who remained stable (81.6% vs. 57.8% and 46.9% vs. 24.4%, *p* < 0.05). Multivariate logistic regression analysis revealed that both hyperuricemia (odds ratio 2.741, 95% CI 1.014–7.413, *p* = 0.047) and intravenous fat emulsion therapy (odds ratio 2.645, 95% CI 1.046–6.686, *p* = 0.040) were independent predictors of gout flares. Furthermore, gout attacks occurred sooner in patients receiving intravenous fat emulsion therapy compared to those not receiving it (median: 4 days (interquartile range: 2) vs. median: 5 days (interquartile range: 2.25), *p* = 0.049).

**Conclusion:**

Our study revealed a high incidence of gout flares during episodes of active gastrointestinal bleeding, with patients undergoing intravenous fat emulsion therapy and those with hyperuricemia being at increased risk.

## Introduction

Gout is one of the most prevalent forms of inflammatory arthritis, affecting approximately 1.1 to 6.8% of the global population [1, 2]. It is now understood that gout arises due to the deposition of monosodium urate (MSU) crystals on cartilages or entheses [3]. The hallmark symptoms of a gout flare encompass redness, swelling, warmth, and excruciating pain in the joints, notably in the lower extremities. While a gout flare is often self-limiting and typically resolves within approximately 14 days during its early stages, it can evolve into a chronic condition as it progresses. The application of non-steroidal anti-inflammatory drugs (NSAIDs), colchicine, or corticosteroids, either alone or in combination, has proven effective in managing acute inflammation associated with gout.

Individuals with gout face an elevated risk of being hospitalized. Inpatient gout flares are common and will add 3–6 days to an admission [4]. A number of studies have elaborated on the risk factors for gout flares during in hospitalization [5–9]. These risk factors fall mainly into the following four categories: (1) Serum uric acid: Both hyperuricemia and fluctuations in serum uric acid are associated with gout attacks [6–9]. Consistently, overhydrate and agents that affect serum uric acid levels such as urate-lowering therapy (ULT) and diuretics and are also relate to the gout flares [6]. Long-term (over 3 months) ULT reduces the risk of gout attacks, while short-term (within 3 months) ULT increases it [6, 9]. (2) Gout prophylaxis: Colchicine prophylaxis decreases the risk of gout flares [7, 8], whereas gout prophylaxis started or increased in the reverse direction, a phenomenon that may be explained by concurrently initiated or adjusted ULT [6]. (3) Complications of gout: Tophus and kidney injury are connected with gout attacks [6]. (4) Surgery: Surgery, particularly cancer-related and abdominal surgery, as well as postsurgical total parenteral nutrition, are also linked to gout flares [7, 8].

As previously mentioned, anti-inflammatory therapies like NSAIDs, colchicine, or glucocorticoids are vital in the management of gout flares. Despite their efficacy, a common drawback of these drugs is the potential for gastrointestinal adverse effects, including diarrhea, vomiting, peptic ulcers, and, in severe cases, gastrointestinal bleeding. Research indicates that the incidence of upper gastrointestinal hemorrhage among gout patients using non-selective NSAIDs is 7.2% [10]. Moreover, a study investigating the relationship between short-term glucocorticoid use and peptic ulcer bleeding found that 6% of patients administered glucocorticoids for a week experienced peptic ulcer bleeding [11]. Although gastrointestinal adverse events like diarrhea, vomiting, and nausea are frequently observed during colchicine treatment, even at a dose of 0.5 mg once daily, the incidence of gastrointestinal bleeding does not appear to rise significantly [12]. However, there is a significant lack of large-scale studies examining the rate of gastrointestinal hemorrhage associated with therapeutic doses used for acute gout.

Acute gout attacks are frequently encountered in individuals experiencing active gastrointestinal bleeding, posing a formidable challenge due to the high incidence of adverse effects stemming from anti-inflammatory therapies. However, limited research exists on the risk factors contributing to gout flares during episodes of active gastrointestinal bleeding. Consequently, this study aims to provide an in-depth analysis of the clinical characteristics and risk factors associated with gout flares in the context of active gastrointestinal bleeding.

## Methods

### Study design and participants

**Fig. 1:**
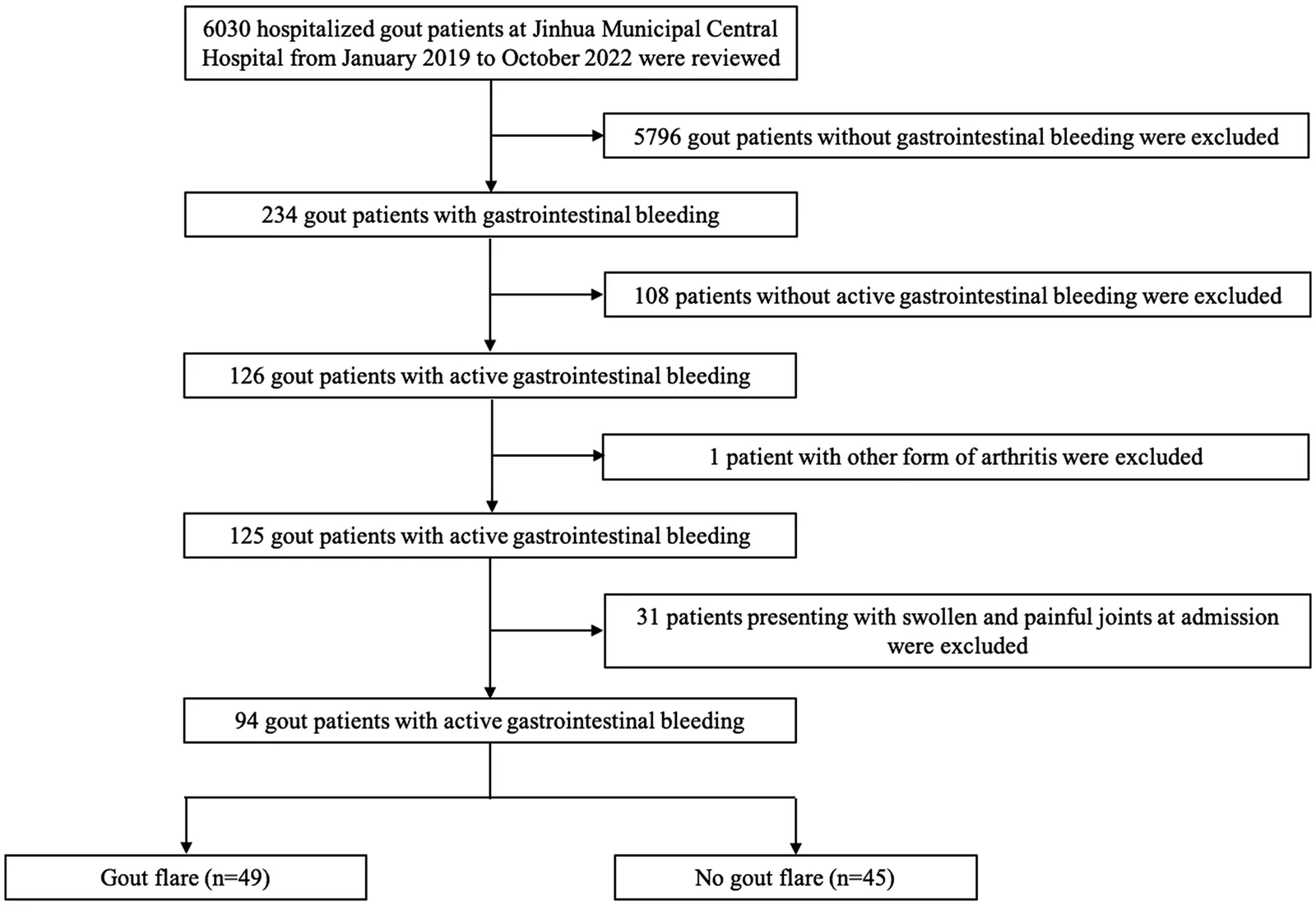
Flow chart of the study. 6030 hospitalized patients diagnosed as gout were reviewed, and based on the study criteria, 94 cases were ultimately included in the analysis

This retrospective observational study was conducted exclusively at Jinhua Municipal Central Hospital from January 2019 to October 2022. Eligible participants for this study included patients who meeting the 2015 ACR/EULAR classification criteria for gout and concurrently experienced active gastrointestinal bleeding upon enrollment. Notably, patients presenting with swollen and painful joints at admission were excluded. Individuals with other forms of arthritis were also excluded from the study. A flow chart of this study is shown in Fig. 1. All of the patients included suffered from gout flares after gastrointestinal bleeding.

Ethical considerations were rigorously adhered to in accordance with the Declaration of Helsinki principles, and the study obtained ethical approval from the Ethics Committee of Jinhua Municipal Central Hospital (Approval No. [2022]366)

### Clinical data collection

Clinical data was collected from electronic medical records according to the International Classification of Diseases (ICD) 11th revision code for gout and gastrointestinal bleeding, and was in full compliance with the protocols established by Jinhua Municipal Central Hospital. The dataset encompassed a range of variables, including age, gender, medical history, body mass index (BMI), comorbidities (such as hypertension, diabetes, renal insufficiency, heart failure, cardiovascular and cerebrovascular events, and cirrhosis), prior usage of uric acid-lowering medications and diuretics prior to admission, serum uric acid levels, hemoglobin levels, instances of blood transfusion, utilization of endoscopic procedures, intravenous fat emulsion therapy, length of hospital stays, recurrent bleeding episodes, gout attacks, and specific details pertaining to the gout attacks (including the site of flare, day of hospitalization, and the administration of anti-inflammatory drugs, among others). The intravenous fat emulsions included C8-24, C6-24, and C14-24, administered at a daily dose of 250 ml. Large amount of missing data resulted in the exclusion of information on tophus and fluid intake.

### Definitions of gout flare and active gastrointestinal bleeding

In this study, a gout flare was defined as a clinically evident episode of acute inflammation induced by monosodium urate crystals, a definition endorsed by the Gout, Hyperuricemia and Crystal-associated Disease Network (G-CAN) [13]. To be specific, all patients met at least 3 of the following criteria: (1) Patient-defined gout flare; (2) Pain at rest score >3 on a 0–10 numerical rating scale; (3) Presence of at least 1 swollen joint; (4) Presence of at least 1 warm joint [14]. The gout flares were confirmed by the rheumatologist. Besides, active gastrointestinal bleeding was defined based on the following criteria: (1) Hematemesis or hematochezia within the 24-h period preceding admission; (2) A positive result on the fecal occult blood test; (3) Absence of bleeding in the oral cavity and respiratory system.

### Outcomes

The primary outcome measure in this study was the incidence of gout flares among patients experiencing active gastrointestinal bleeding during their hospitalization. For subsequent analysis, patients were categorized into two distinct groups: the “flare group” and the “stable group”.

### Statistical analysis

Statistical analyses were conducted using SPSS software (version 26). Normality testing was performed using the Kolmogorov–Smirnov test. Normally distributed continuous variables were presented as mean ± SD and analyzed using the Student’s *t*-test, whereas non-normally distributed continuous variables were represented as median (interquartile range) and assessed via the Mann-Whitney test. Categorical data comparisons were conducted using the Pearson chi-square tests. Risk factor analysis was carried out using multivariate logistic regression, focusing on variables with *P* values either below 0.10 in the univariate analysis or deemed clinically significant. Hyperuricemia, ULT, intravenous fat emulsion, endoscopic procedure and transfusion were finally included as dependent variables in the model based on the above criterion. Statistical significance was determined at a threshold of *p* < 0.05.

## Results

94 individuals meeting the above-mentioned criteria were included in the study. As detailed in Table 1, 52.1% (n = 49/94) experienced gout flares. Notably, there were no significant differences in age, disease duration, or comorbidity profiles between the two groups. However, the proportion of individuals with hyperuricemia (serum uric acid levels > 420 μmol/L) and those subjected to intravenous fat emulsion therapy was significantly higher within the flare group when compared to the stable group. Additionally, patients who encountered gout flares during their hospitalization exhibited a statistically significant longer median length of stay (10 [5] days vs. 8 [5] days, *p* = 0.023) compared to the stable group.

**Table 1:** Clinical characteristics

	Flare (n = 49)	Stable (n = 45)		*P* value
Gender (male)	46/49, 93.9%	43/45, 95.6%	X^2^ = 0.000	>0.999
Age (years)	64.65 ± 12.93	67.67 ± 11.82	t = 1.176	0.243
Duration of gout > 10 years	23/49, 46.9%	24/45, 53.3%	X^2^ = 0.384	0.536
Body Mass Index > 24.0 kg/m^2^	15/49, 30.6%	19/45, 42.2%	X^2^ = 1.370	0.242
Comorbidity				
Hypertension	20/49, 40.8%	25/45, 55.6%	X^2^ = 2.042	0.153
Diabetes	7/49, 14.3%	6/45, 13.3%	X^2^ = 0.018	0.894
Coronary heart disease	7/49, 14.3%	7/45, 15.6%	X^2^ = 0.030	0.863
Cerebral infarction	2/49, 4.0%	3/45, 6.7%	X^2^ = 0.010	0.922
Heart failure	4/49, 8.2%	3/45, 6.7%	X^2^ = 0.000	>0.999
Renal insufficiency	25/49, 51.0%	21/45, 46.7%	X^2^ = 0.178	0.673
Cirrhosis	10/49, 20.4%	11/45, 24.4%	X^2^ = 0.220	0.639
Alcohol consumption	27/49, 55.1%	20/45, 44.4%	X^2^ = 1.066	0.302
Serum uric acid (μmol/L)	535.00 (163.50)	487.82 ± 170.86	z = −1.582	0.114
Hyperuricemia	40/49, 81.6%	26/45, 57.8%	X^2^ = 6.382	**0.012**
Urate-lowering therapy	4/49, 8.2%	6/45, 13.3%	X^2^ = 0.228	0.633
Hemoglobin (g/L)	71 (20)	80.44 ± 18.43	Z = −1.045	0.296
Hemoglobin < 70 g/L	20/49, 40.8%	15/45, 33.3%	X^2^ = −0.562	0.453
Diuretic	10/49, 20.4%	8/45, 17.8%	X^2^ = 0.105	0.746
Causes of bleeding				
Peptic ulcer	26/49, 53.1%	23/45, 51.1%	X^2^ = 1.094	0.296
Esophageal variceal bleeding	7/49, 14.3%	7/45, 15.6%	X^2^ = 0.030	0.863
Others and unknown	16/49, 32.7%	15/45, 33.3%	X^2^ = 0.005	0.944
Length of stay	10 (5)	8 (5)	Z = −2.279	**0.023**
Intravenous fat emulsion	23/49, 46.9%	11/45, 24.4%	X^2^ = 5.141	**0.023**
Endoscopic procedure	36/49, 73.4%	32/45, 71.1%	X^2^ = 0.065	0.798
Blood transfusion	30/49, 61.2%	19/45, 42.2%	X^2^ = 3.394	0.065

*Note* Statistically significant results are indicated in bold

The median time interval for the onset of a gout flare was 4 (3) days, with a median involvement of 2 (2) joints. Notably, most patients received treatment with diclofenac diethylamine emulgel, as outlined in Table 2. Among the patients experiencing gout flares, two cases of recurrent bleeding were observed. One patient received a daily dose of 5 mg of dexamethasone, while the other was administered 1.5 mg of colchicine per day. Interestingly, two instances of recurrent bleeding were reported among the stable group of patients.

**Table 2:** Characteristics of gout flare

Variables	n = 49		
Day of hospitalization	4 (3)		
Number of involved joint	2 (2)		
Flare site			
Upper extremity	0 (1)	Z = −4.053	***P* < 0.001**
Lower extremity	1 (1)		
Drugs			
Diclofenac diethylamine emulgel	44/49, 89.8%		
Colchicine	6/49, 12.2%		
Cyclooxygenase-2 inhibitor	7/49, 14.3%		
Glucocorticoids	18/49, 36.7%		
Diclofenac sodium suppositories	15/49, 30.6%		

*Note* Statistically significant results are indicated in bold

During the univariate analysis, hyperuricemia (odd ratio (OR) 3.248, *P* = 0.013) and the use of intravenous fat emulsion (OR 2.734, *P* = 0.025) were significantly associated with the occurrence of gout flares during gastrointestinal bleeding, as indicated in Table 3. After adjusting for factors including urate-lowering therapy, transfusion, and endoscopic procedures, hyperuricemia (OR 2.741, *P* = 0.047) and intravenous fat emulsion therapy (OR 2.645, *P* = 0.040) were identified as independent factors that increased the risk of acute gout flares. Importantly, there was no significant interaction between hyperuricemia and intravenous fat emulsion therapy (*p*_interaction_ = 0.226).

**Table 3:** Risk factors of gout flare

	OR	95% CI	*P* value	Adjusted OR	95% CI	*P* value
Hyperuricemia	3.248	1.276–8.267	**0.013**	2.741	1.014–7.413	**0.047**
Cirrhosis	0.793	0.300–2.095	0.639			
Urate-lowering therapy	0.578	0.152–2.197	0.421	0.853	0.187–3.891	0.837
Diuretic	1.186	0.422–3.331	0.746			
Intravenous fat emulsion	2.734	1.132–6.602	**0.025**	2.645	1.046–6.686	**0.040**
Endoscopic procedure	1.125	0.455–2.779	0.799	1.032	0.390–2.731	0.950
Alcohol consumption	1.534	0.680–3.462	0.303			
Hemoglobin < 70 g/L	1.379	0.594–3.201	0.454			
Transfusion	2.161	0.947–4.929	0.067	2.001	0.828–4.834	0.123

*Note* Statistically significant results are indicated in bold

As presented in Table 4, within the group of patients experiencing gout attacks, a total of 23 individuals were administered intravenous fat emulsion therapy. It is noteworthy that gout flares manifested earlier in those who received intravenous fat emulsion therapy compared to those who did not (4 [2] days vs. 5 [2.25] days, *p* = 0.049).

**Table 4:** Comparison of features of gout attack between the intravenous fat emulsion group and the non-intravenous fat emulsion group

	Intravenous fat emulsion (n = 23)	Non-intravenous fat emulsion (n = 26)		
**Initiating intravenous fat emulsion day**				
Day 1	19			
Day 2	3			
Day 3	1			
Time to Gout Flare (Days after Hospitalization)	4 (2)	5 (2.25)	Z = −1.968	**0.049**
Time to Gout Flare (Days after intravenous fat emulsion)	4 (3)			
Number of involved joint	2 (2)	2 (2)	Z = −0.136	0.892
**Flare site**				
Upper extremity	0 (1)	0 (1)	Z = −0.707	0.480
Lower extremity	2 (1)	1 (1)	Z = −0.656	0.512

*Note* Statistically significant results are indicated in bold

## Discussion

Our study highlights that hyperuricemia and the administration of intravenous fat emulsion are associated with an increased risk of acute gout attacks in patients with gout who also have active gastrointestinal bleeding.

Over the years, many studies investigating the risk factors for gout attacks have consistently identified hyperuricemia as a significant risk factor [7, 15]. Indeed, hyperuricemia is known to promote the formation of monosodium urate crystals in the joints. Additionally, uric acid-lowering therapy has been shown to protect against gout attacks [16]. While our study findings yielded consistent findings, it is important to note that statistical significance could not be reached due to the low utilization rate of urate-lowering medications and the relatively small sample size in our study. Nevertheless, we strongly advocate for the use of urate-lowering therapy to achieve target uric acid levels in patients with gout.

Our study provides hitherto undocumented evidence of an association between intravenous fat emulsion therapy and gout attacks, and we speculated that fat emulsion injections may contribute to flare-ups of gout. In the context of gastrointestinal hemorrhage, fasting is a crucial measure to prevent further damage from gastric acid and digestive enzymes, necessitating parenteral nutrition to supply essential fluids, electrolytes, and nutrients. During fasting, intravenous fat emulsion serves as the primary source of fatty acids administered via parenteral nutrition. Fat emulsion elevates free fatty acid levels in patients [17], and these free fatty acids serve as the initial signal for the Nod-like receptor family pyrin domain containing 3 activation [18], a process pivotal in gout pathogenesis. Therefore, we hypothesize that intravenous fat emulsion may promote gout flares by increasing fatty acid levels. Indeed, a study by Pei et al. demonstrated higher serum free fatty acid levels in patients experiencing acute gout attacks compared to stable patients, while no significant differences were observed in triglyceride and low-density lipoprotein levels [19]. Consequently, it is essential to avoid intravenous fat emulsion in patients with gout who also have active gastrointestinal bleeding, opting instead for early enteral nutrition, as it does not demonstrate a significantly higher risk of rebleeding or mortality when compared to delayed enteral nutrition [20]. However, it should be noted that the intravenous fat emulsion in our regression model was only marginally significant. Moreover, a causal relationship cannot be concluded by a simple logistic regression model.

Given that topical anti-inflammatory analgesics exhibited limited ability to effectively treat acute gout attacks, systemic anti-inflammatory drugs are often used. Given the potential risk of gastrointestinal bleeding associated with systemic anti-inflammatory drugs, the incidence of rebleeding becomes a key concern. In our study, four cases of rebleeding were observed, with two occurring in each of the gout flare and stable groups, suggesting that systemic anti-inflammatory therapy may be relatively safe for patients with gastrointestinal bleeding. However, it is important to acknowledge the small sample size of our study, emphasizing the need for larger studies to evaluate the overall effect. Interestingly, one study revealed a higher rebleeding incidence in patients with duodenal ulcers [21], warranting heightened attention in this specific patient group. Combining celecoxib with a proton pump inhibitor (PPI) may significantly reduce the risk of gastrointestinal rebleeding in high-risk patients when compared to non-selective NSAIDs, though caution is advised in patients with renal insufficiency [22]. However, limited research has focused on the use of glucocorticoids and colchicine in relation to the incidence of gastrointestinal rebleeding, and further studies are warranted to determine safe anti-inflammatory regimens for patients with gout and acute gastrointestinal bleeding.

Our study has two major limitations. Firstly, it is a retrospective study and is susceptible to inherent biases and limitations associated with such designs. Secondly, the sample size in our study was relatively small. Lastly, some significant variables reported in other relevant articles were not included in the logistic regression model due to data missing. Consequently, larger, high-quality randomized controlled trials are necessary to corroborate the relationship between intravenous fat emulsion and gout attacks and to establish the optimal dosage.

## Conclusion

Hyperuricemia and intravenous fat emulsion are risk factors for gout flare during active gastrointestinal bleeding. Controlling serum uric acid levels and avoiding intravenous fat emulsion therapy can help prevent gout attacks in the context of active gastrointestinal hemorrhage.

## Data Availability

Data sharing is not applicable to this article as no datasets were generated or analysed during the current study.

## List of abbreviations

MSU: Monosodium urate
NSAIDs: Non-steroidal anti-inflammatory drugs
ULT: Urate-lowering therapy
G-CAN: Gout, Hyperuricemia and Crystal-associated Disease Network
BMI: Body mass index
OR: Odd ratio
PPI: Proton pump inhibitor
